# Genome-Wide Association Study Reveals Genetic Markers for Antimicrobial Resistance in Mycoplasma bovis

**DOI:** 10.1128/Spectrum.00262-21

**Published:** 2021-10-06

**Authors:** Jade Bokma, Nick Vereecke, Hans Nauwynck, Freddy Haesebrouck, Sebastiaan Theuns, Bart Pardon, Filip Boyen

**Affiliations:** a Department of Large Animal Internal Medicine, Faculty of Veterinary Medicine, Ghent University, Merelbeke, Belgium; b Department of Pathology, Bacteriology, and Avian Diseases, Faculty of Veterinary Medicine, Ghent University, Merelbeke, Belgium; c Department of Virology, Parasitology, and Immunology, Faculty of Veterinary Medicine, Ghent University, Merelbeke, Belgium; d PathoSense BV, Lier, Belgium; University of Pittsburgh School of Medicine

**Keywords:** epidemiological cutoff, fluoroquinolones, gamithromycin, gentamicin, macrolides, Nanopore sequencing

## Abstract

Mycoplasma bovis causes many health and welfare problems in cattle. Due to the absence of clear insights regarding transmission dynamics and the lack of a registered vaccine in Europe, control of an outbreak depends mainly on antimicrobial therapy. Unfortunately, antimicrobial susceptibility testing (AST) is usually not performed, because it is time-consuming and no standard protocol or clinical breakpoints are available. Fast identification of genetic markers associated with acquired resistance may at least partly resolve former issues. Therefore, the aims of this study were to implement a first genome-wide association study (GWAS) approach to identify genetic markers linked to antimicrobial resistance (AMR) in M. bovis using rapid long-read sequencing and to evaluate different epidemiological cutoff (ECOFF) thresholds. High-quality genomes of 100 M. bovis isolates were generated by Nanopore sequencing, and isolates were categorized as wild-type or non-wild-type isolates based on MIC testing results. Subsequently, a k-mer-based GWAS analysis was performed to link genotypes with phenotypes based on different ECOFF thresholds. This resulted in potential genetic markers for macrolides (gamithromycin and tylosin) (23S rRNA gene and 50S ribosomal unit) and enrofloxacin (GyrA and ParC). Also, for tilmicosin and the tetracyclines, previously described mutations in both 23S rRNA alleles and in one or both 16S rRNA alleles were observed. In addition, two new 16S rRNA mutations were possibly associated with gentamicin resistance. In conclusion, this study shows the potential of quick high-quality Nanopore sequencing and GWAS analysis in the evaluation of phenotypic ECOFF thresholds and the rapid identification of M. bovis strains with acquired resistance.

**IMPORTANCE** Mycoplasma bovis is a leading cause of pneumonia but also causes other clinical signs in cattle. Since no effective vaccine is available, current M. bovis outbreak treatment relies primarily on the use of antimicrobials. However, M. bovis is naturally resistant to different antimicrobials, and acquired resistance against macrolides and fluoroquinolones is frequently described. Therefore, AST is important to provide appropriate and rapid antimicrobial treatment in the framework of AMR and to prevent the disease from spreading and/or becoming chronic. Unfortunately, phenotypic AST is time-consuming and, due to the lack of clinical breakpoints, the interpretation of AST in M. bovis is limited to the use of ECOFF values. Therefore, the objective of this study was to identify known and potentially new genetic markers linked to AMR phenotypes of M. bovis isolates, exploiting the power of a GWAS approach. For this, we used high-quality and complete Nanopore-sequenced M. bovis genomes of 100 isolates.

## INTRODUCTION

Mycoplasma bovis is an important veterinary pathogen causing various diseases in cattle, such as pneumonia, mastitis, and arthritis (1, 2). Transmission pathways and pathophysiology are not fully understood, hampering development of effective prevention and control (3, 4). Also, no effective commercial vaccine is available. Therefore, the most important way to control an outbreak of M. bovis-associated disease remains the adequate use of antimicrobials (3). M. bovis is naturally resistant to β-lactam antibiotics and (potentiated) sulfonamides (5). Given that the use of critically important fluoroquinolones as first-intention treatment in animals is strongly discouraged (6), empirical therapy is mainly limited to florfenicol (FLOR), tetracyclines, and macrolides (7, 8).

Worldwide, an overall increase in acquired resistance in M. bovis for mostly macrolides and tetracyclines but also for FLOR, lincosamides, and fluoroquinolones is reported (7, 9–13). To rationalize antimicrobial use to treat M. bovis infections, there is an urgent need for a rapid and meaningful antibiogram. Unfortunately, phenotypic antimicrobial susceptibility testing (AST) of M. bovis is time-consuming (up to 2 weeks), difficult to compare between studies because no standard protocol is available, and almost impossible to translate into *in vivo* results, considering the absence of M. bovis-specific clinical breakpoints (CBPs). Therefore, phenotypic AST is not routinely used in practice.

A genetic approach may at least partly resolve former issues, since it is faster and more standardized for AST in M. bovis (14, 15). Molecular detection of antimicrobial resistance (AMR) determinants with methods based on targeted PCR was explored in the past for M. bovis (16, 17), and targeted gene sequencing for macrolide resistance has already been implemented in research and development settings for Mycoplasma pneumoniae community-acquired infections in humans (18). Recently, the association between point mutations identified with whole-genome sequencing and phenotypic AMR have been explored in specific regions of three M. bovis strains (19) and for macrolides with a large set of isolates (15). However, these targeted approaches may result in a narrowed view, and potential new genomic alterations within genes, operons, or even promoter, enhancer, and/or inhibitory regions might be overlooked (15, 20). One way to overcome this shortcoming is the use of a genome-wide association study (GWAS) to confirm the relevance of previously described mutations and to reveal novel associations between genotype and phenotype (21). In addition, this approach can suggest undescribed resistance mechanisms (e.g., through DNA methylation or transcription regulation) in case a whole genome versus phenotype association remains inconclusive. Key to this kind of analysis is the generation of complete and highly accurate bacterial genomes. While short-read sequencing approaches have been typically considered the gold standard for sequence accuracy, they result in highly contiguous genome assemblies for M. bovis due to its distinct genomic architecture. This is mainly due to a low GC content (29.3%), many highly repetitive regions, and the use of a distinct genetic code (translation table 4) (22). High-quality (complete and accurate) long-read sequencing approaches have been shown to be promising for all-in-one diagnostic workflows (including identification, strain typing, and possibly AMR detection), enormously reducing costs and turnaround times (23, 24). The aim of this study was to identify known and potentially new genetic markers linked with AMR phenotypes in a collection of 100 M. bovis isolates, exploiting the power of a GWAS approach with high-quality and complete Nanopore-sequenced M. bovis genomes.

## RESULTS

### Phenotypic AST and the evaluation of high-quality M. bovis genomes.

Results of the phenotypic AST, resulting in the determination of the epidemiological cutoff (ECOFF) values, were published elsewhere (13). All M. bovis PG45 tests showed comparable MIC values, classifying them in the wild-type (WT) population for each antimicrobial tested. Only high-quality and complete genomes (*n* = 95 of 100 genomes) were included in the GWAS; therefore, a total of 5 genomes were excluded from subsequent GWAS analyses. An overview of all genomes, accession numbers, and quality assessment can be found in Table S1 in the supplemental material. Classification of M. bovis strains into (non-)WT or susceptible/resistant isolates was based on the ECOFFs determined previously by Bokma and colleagues (13). Results for the 95 isolates and M. bovis PG45 in this study are shown in Table 1.

**TABLE 1:** Distribution of antimicrobial susceptibility of 95 Nanopore-sequenced M. bovis isolates and M. bovis PG45 according to ECOFF method (visual estimation, NRI, or ISM)^*a*^

Antimicrobial	Visual estimation			NRI^*b*^			ISM^*b*^ (95%/99%)		
	ECOFF (μg/ml)	No.		ECOFF (μg/ml)	No.		ECOFF (μg/ml)^*c*^	No.	
		WT	Non-WT		WT	Non-WT		WT	Non-WT
FLOR	>16	91	4	>16	91	4	>8/16 (+)	89/91	6/4
OTC	>8	95	0	>8	95	0	>4/8 (+)	93/95	2/0
DOX	>4	94	0	>2	91	3	>1/2 (+)	82/91	12/3
TIL	ND			>1,024			ND		
TYL	>32	46	50	>128^*d*^	46	50	ND		
GAM	>64	53	43	>128^*d*^	56	40	ND		
GEN	>16	95	1	>8	94	2	>4/4 (−)	91/91	5/5
TIA	>0.5	90	3	>0.125	78	15	>0.06/0.06 (−)	59/59	34/34
ENRO	>2	85	8	>1	83	10	>1/2 (±)	83/85	10/8

a Determinations of ECOFFs were published previously (13).

b NRI, normalized resistance interpretation; ISM, iterative statistical method.

c Plots for residuals were checked and categorized as either good fit (+), poor fit (±), or no fit (−), corresponding to whether the subset values are reliable or not. ND, not possible to determine.

d Tentative estimate, because the standard deviation was >1.2 log_2_.

### GWAS analysis based on different ECOFF methods.

First, the GWAS analysis was applied to the different distributions of WT and non-WT isolates based on ECOFFs determined by visual estimation, normalized resistance interpretation (NRI), 95% iterative statistical method (ISM), and 99% ISM approaches (Table 1). The most significant (*P* and *q* values) results for enrofloxacin (ENRO) and tylosin (TYL) were seen when ECOFF was based on the visual estimation method (see Table S2). For gamithromycin (GAM), a negligible difference between the visual estimation and NRI results was observed (see Table S2). Unfortunately, for FLOR, oxytetracycline (OTC), doxycycline (DOX), tilmicosin (TIL), gentamicin (GEN), and tiamulin (TIA), the GWAS analysis was not successful because either no or too few (*n* < 5) strains were assigned to the (non-)WT group or no clear association could be made between the classified genotypes and the observed phenotypes. Because not all methods could be applied to all macrolides and the results of the visual estimation were for the most part more significant than those of the other methods, the GWAS results shown below are based on ECOFFs determined by the visual estimation method, as described by Bokma et al. (13).

### Mutations in the M. bovis *gyrA* and *parC* genes are associated with ENRO resistance.

**FIG 1:**
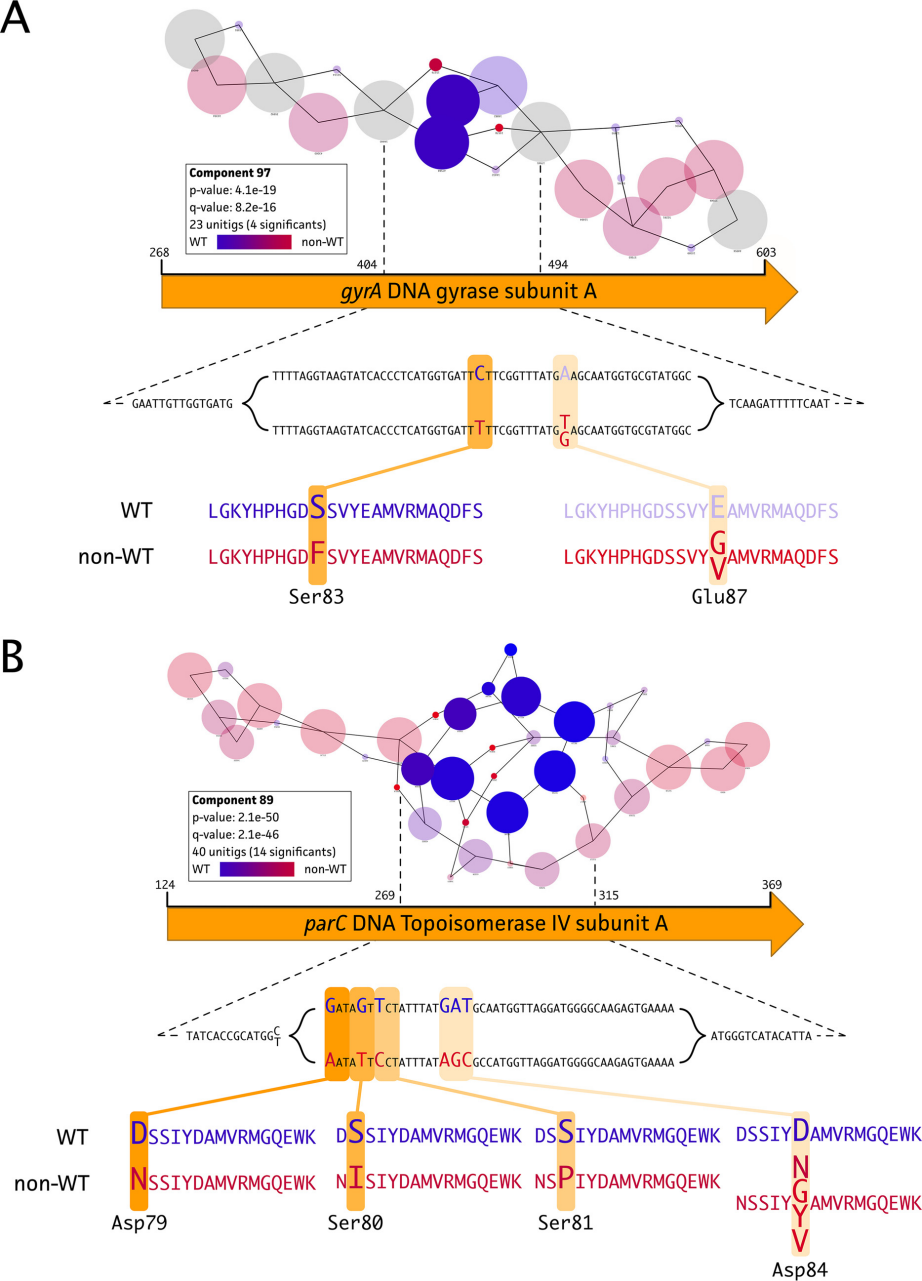
DBGWAS analysis of ENRO resistance in 95 Belgian M. bovis isolates and M. bovis PG45. Significant associations between the ENRO non-WT phenotype (*n* = 8) and genotype could be found for two known fluoroquinolone gene targets, i.e., the *gyrA* (A) and *parC* (B) genes. Further in-depth analysis identified two and four nonsynonymous mutations in the GyrA and ParC protein, respectively. Amino acid positions are labeled according to classic Escherichia coli numbering.

**FIG 2:**
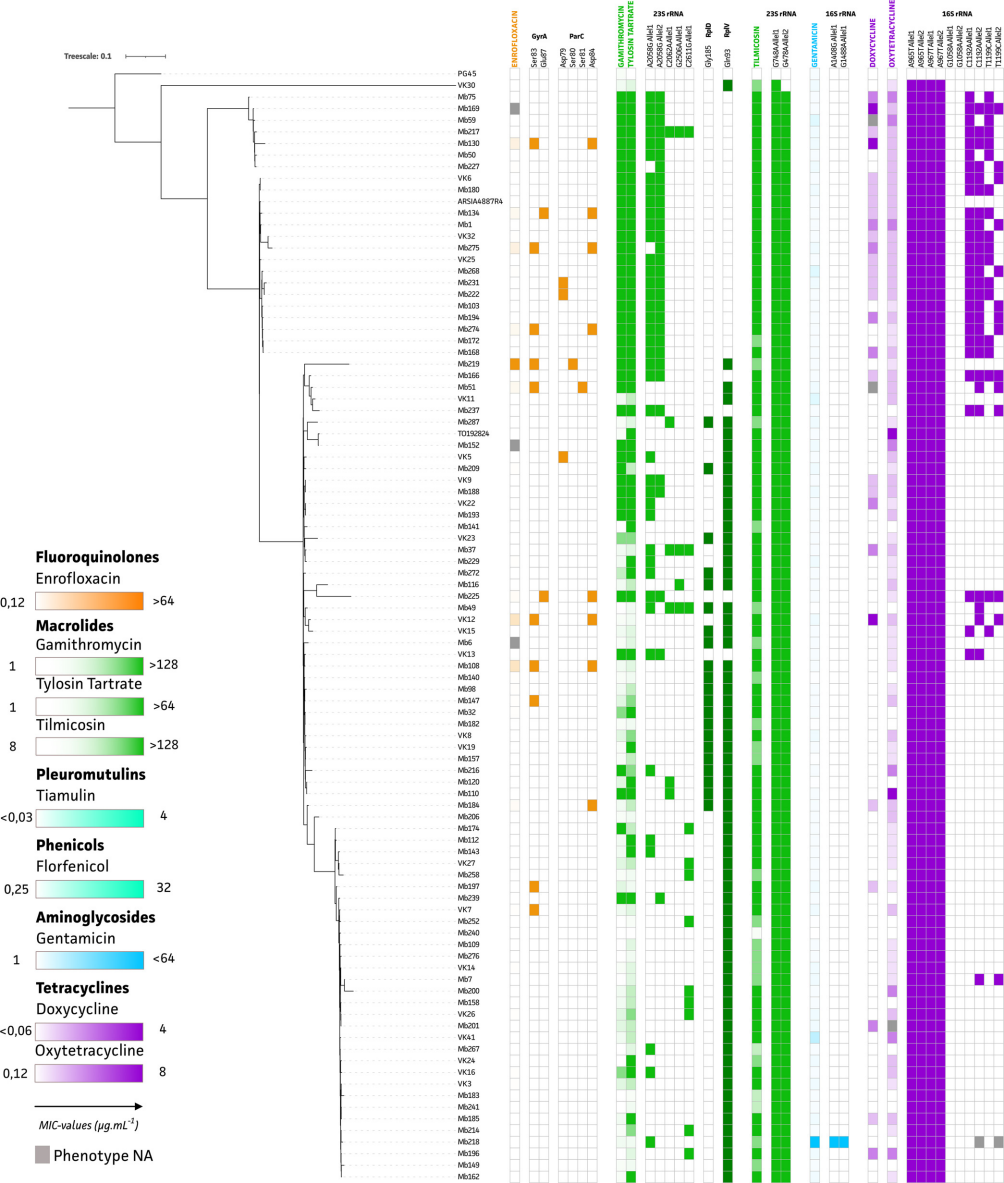
Distribution of phylogenetic tree, MIC values, and (nonsynonymous) mutations of 95 Belgian M. bovis field isolates and M. bovis PG45. The color gradient is corresponding with the MIC values for ENRO (orange), macrolides (GAM, TYL, and TIL) (green), GEN (blue), and tetracyclines (DOX and OTC) (purple), while colored blocks show the presence/absence of (nonsynonymous) mutations. Nucleotide and amino acid positions are labeled according to classic E. coli numbering.

A successful GWAS analysis for the fluoroquinolone ENRO could be performed because the non-WT population contained 8 isolates of 96 (95 field isolates plus PG45) total high-quality genomes. Two significant components were identified, covering the *gyrA* (Fig. 1A) and *parC* (Fig. 1B) gene targets, for the ENRO phenotype. These genes encode the DNA gyrase subunit A and the DNA topoisomerase 4 subunit A protein, respectively. Other components were analyzed but did not show an association with the ENRO-resistant phenotype. In-depth analysis of these “suspected” target genes from each genome showed the existence of two nonsynonymous mutations (Ser83Phe and Glu87Gly/Val) located in the quinolone-resistance-determining region (QRDR). In addition, four possible mutations (Asp79Asn, Ser80Ile, Ser81Pro, and Asp84Asn/Tyr/Val/Gly) were identified in the ParC protein (Fig. 2, orange).

**FIG 3:**
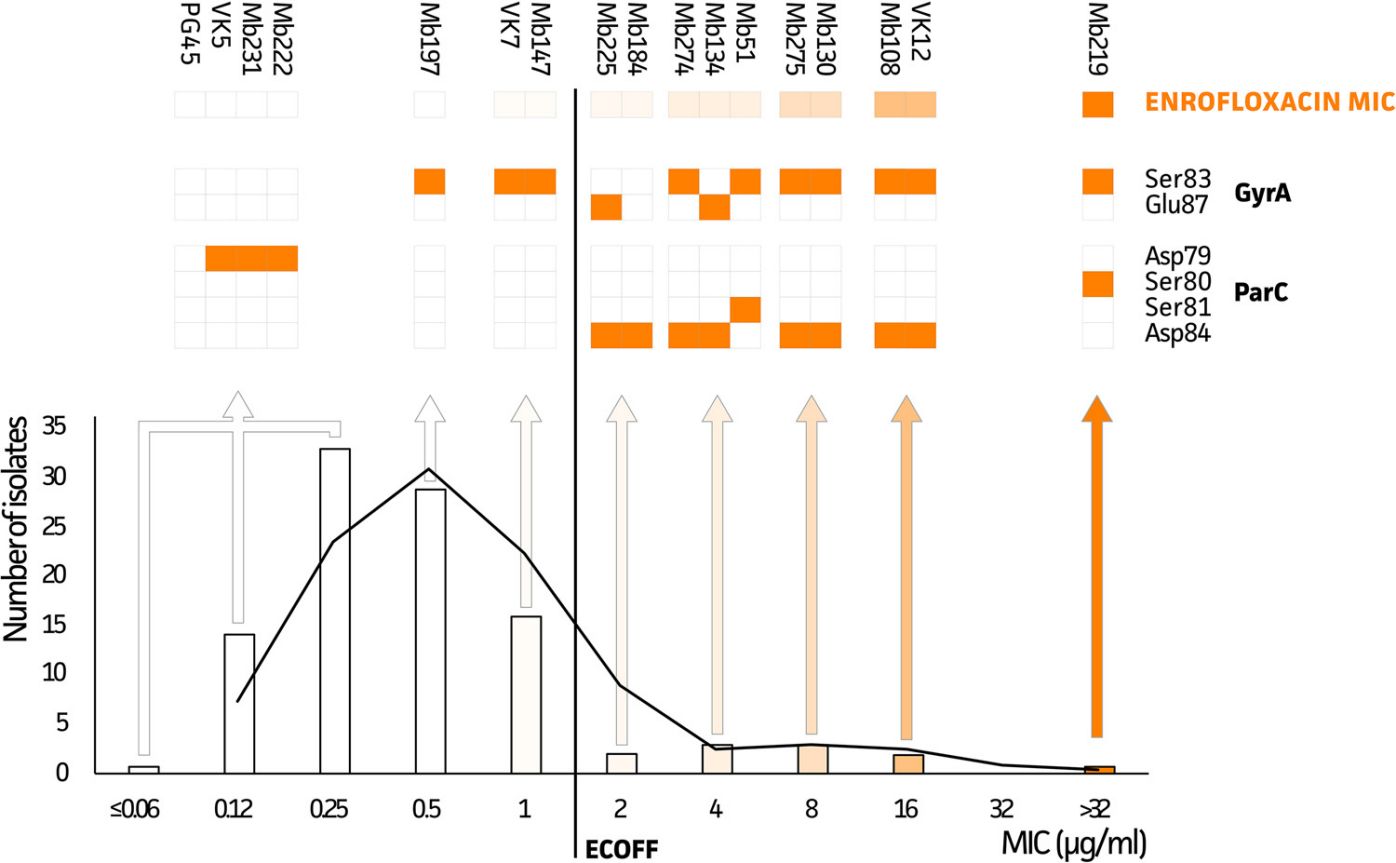
Distribution of MIC values for 100 Belgian M. bovis isolates and M. bovis PG45 and their associated mutations in GyrA and ParC. All strains, except Mb184, with a double mutation in GyrA and ParC, show MIC values above the ECOFF (*n* = 10).

The genotypic data suggested that the ECOFF could be lowered to a MIC of >1 μg/ml since isolate Mb225 (MIC of 2 μg/ml) showed the presence of mutations in both GyrA and ParC proteins. For the other isolate with a MIC of 2 μg/ml (Mb184), only the predominant (8/10 isolates) Asp84 mutation in the ParC protein was observed. Interestingly, based on combinations of these mutations, strains could be classified into different ENRO phenotypes (Fig. 3). While the Ser83Phe mutation in GyrA is predominant (10/16 isolates), its occurrence together with the Ser80Ile mutation in ParC resulted in the highest MIC value (≥32 μg/ml). Strains with other combinations of mutations in both genes (Ser83 or Glu87 in GyrA and Ser81 or Asp84 in ParC) showed MIC values ranging between 2 and 16 μg/ml, with the exception of the Mb184 strain, which only had the Asp84 mutation in the ParC protein. While six strains (Mb147, VK7, Mb197, Mb222, Mb231, and VK5) with MIC values below the newly suggested ECOFF (≤1 μg/ml) harbored a single mutation in either GyrA (Ser83) or ParC (Asp79), all other strains with MIC values below this ECOFF value showed *gyrA* and *parC* genotypes that could not be distinguished from the M. bovis PG45 reference.

### Macrolide resistance in M. bovis is associated with genetic markers in the 23S ribosomal subunit.

Since there was only 1 isolate belonging to the TIL WT population (PG45), a GWAS could not be performed for this antibiotic. However, the determined ECOFF allowed us to identify a known mutation by investigating previously reported resistance target genes. The G748A mutation in domain II of both 23S rRNA alleles was observed in the non-WT population (*n* = 95) and was not present in PG45 (Fig. 2, green). For the two other macrolides, GAM and TYL, 52 and 45 of 96 isolates (95 field isolates plus PG45), respectively, belonged to the non-WT population. The De Bruijn Graph based Genome Wide Association Study (DBGWAS) analysis highlighted the association of both the 23S rRNA gene and the ribosomal operon in the resistance phenotypes of both TYL and GAM. Hence, both 23S rRNA alleles and all 50S accessory ribosomal proteins were extracted and screened for mutations in association with TYL and GAM.

**FIG 4:**
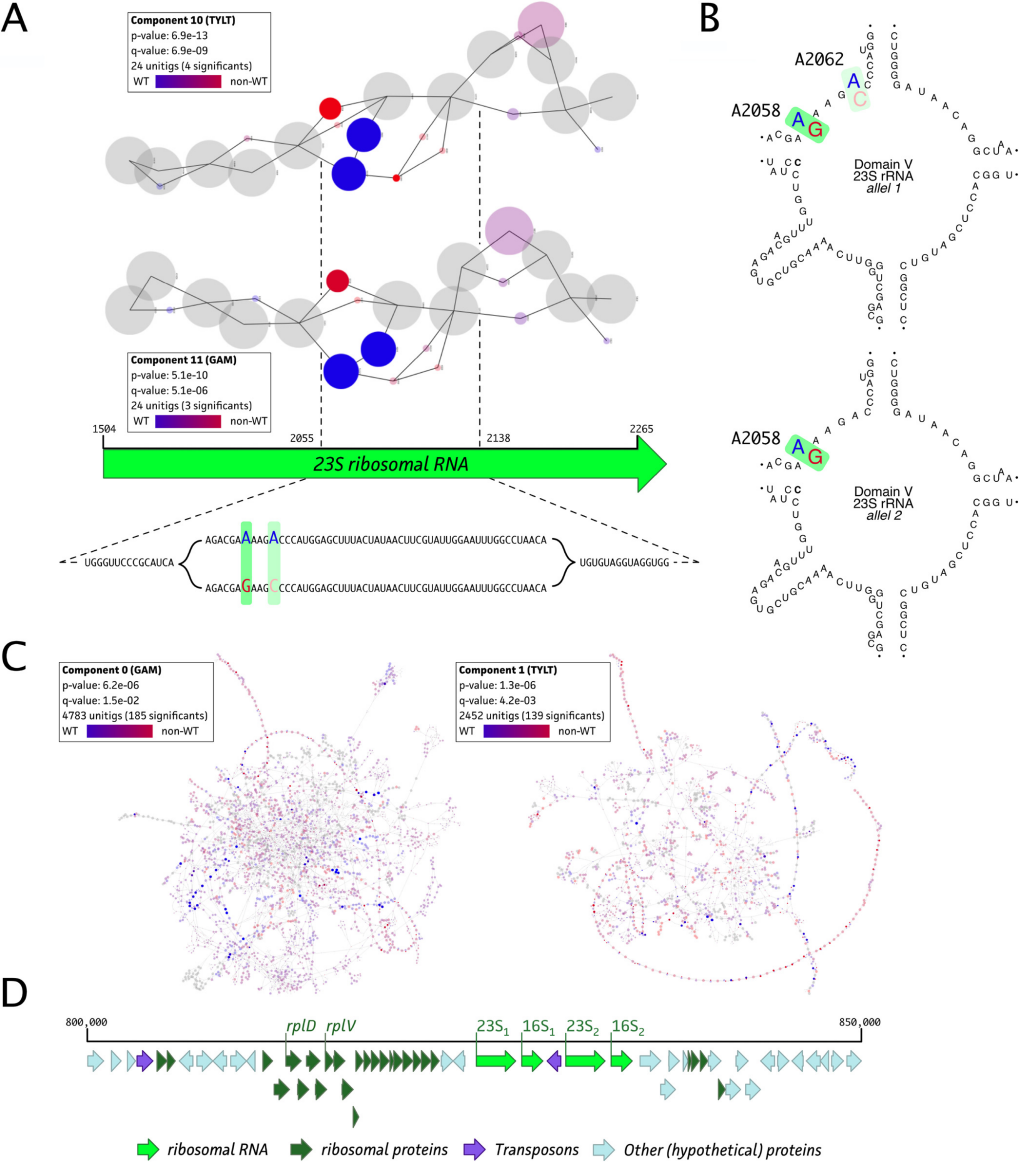
DBGWAS analysis for GAM and TYL resistance in 95 Belgian M. bovis isolates and M. bovis PG45. (A) Association of GAM (non-WT, *n* = 43) and TYL (non-WT, *n* = 50) genotypes with phenotypes resulted in a shared 23S rRNA target association. (B) Secondary structure of domain V of both 23S rRNA alleles, showing the observed mutations (A2058 and A2062). The 23S rRNA positions are labeled according to classic E. coli numbering. (C) DBGWAS analysis output highlights a complex k-mer web, including continuous k-mer strands, suggesting the association of a genetic operon with the phenotype. (D) Genetic context of the M. bovis ribosomal operon, indicating known GAM and TYL target genes (23S rRNA, *rplD*, and *rplV*).

As shown in Fig. 4A, comparable significant associations of point mutations at positions A2058 and A2062 within domain V of the 23S rRNA gene were identified. The A2058 mutation was found in both alleles, whereas the A2062 mutation was found in only one allele of the 23S rRNA gene (Fig. 4B). The 23S rRNA mutations C2062A, G2506A, and C2611G were present in allele 1 of 6, 4, and 12 isolates, respectively, but it was not possible to link them with observed macrolide phenotypes. In addition, both TYL and GAM phenotypes were associated with an operon, as suggested by the thread-like structure in the DBGWAS analysis output (Fig. 4C) (20). The DBGWAS k-mer annotation and further analyses of the resulting operon suggested the association of the ribosomal operon with the GAM and TYL phenotypes (Fig. 4D). Further analyses of the ribosomal genes revealed GAM/TYL resistance-associated mutations in the *rplD* and *rplV* genes, encoding 50S ribosomal proteins 4 (L4) and 22 (L22), respectively. While no clear association of the nonsynonymous Gly185Val/Arg mutation in the L4 protein with the non-WT TYL/GAM phenotypes was identified, the Gln93His mutation in L22 suggested an association with (combined) GAM and TYL non-WT phenotypes. The latter was observed in all isolates (*n* = 35) harboring the transition at the A2058 position in domain V of one or both alleles of the 23S rRNA, of which 27 strains lacked the Gln93His mutation in the L22 ribosomal protein. Still, 6 of 41 doubly TYL- and GAM-resistant isolates showed distinct mutation patterns that could not be linked to a specific resistance phenotype (Fig. 2, green), and 15% of isolates (6/41 isolates) showing a double TYL and GAM resistance phenotype could not be linked to a specific non-WT phenotype (Fig. 2, green). This was also the case for 7 and 13 strains that belonged to only one of GAM or TYL non-WT populations, respectively.

### Mutations in the 16S rRNA possibly associated with tetracycline resistance.

Depending on the ECOFF used, no to limited (13%) phenotypic tetracycline resistance was detected, and no significant associations could be observed in the GWAS study. Nevertheless, previously reported 16S rRNA mutations possibly associated with resistance were detected (Fig. 2, purple). In all isolates, one or more mutations were identified, except for M. bovis PG45 (MIC of ≤0.12 μg/ml), in which no mutations were observed at residue 965, 967, 1058, 1192, or 1199 of the 16S rRNA. In all 95 field isolates, the A965T and A967T mutations are present, with additional mutations C1192A and T1199C (Fig. 2, purple).

### Marker mutation in 16S rRNA observed in a GEN-resistant M. bovis isolate.

The GWAS analysis could not be performed for GEN because only 1 strain with acquired resistance was present in the data set. The M. bovis isolate Mb218 showed a significantly higher MIC (64 μg/ml), compared to the WT population (<32 μg/ml). Since GEN is known to act on the 16S ribosomal subunit, all small ribosomal proteins and both 16S rRNA alleles were manually checked for mutations. Two transversions (A1408G and G1488A) in either one of both 16S rRNA alleles were observed in the Mb218 strain. Because none of the WT isolates harbored these mutations and both A1408 and G1488 transversions were located at or near the GEN binding site of domain II of the 16S rRNA, these mutations are possibly marker mutations for GEN resistance in M. bovis (Fig. 2, blue).

## DISCUSSION

In this study, we exploited a GWAS approach to associate the M. bovis genotype with phenotypic antimicrobial susceptibility test results. High-quality complete and accurate whole genomes were generated using Nanopore sequencing and an optimized taxon-specific base-calling model and assembly as described previously (23). In addition, different methods to determine ECOFFs and thus delineation of the M. bovis WT population and strains with acquired resistance (non-WT strains) were explored. The GWAS analysis showed significant and clear results for the critically important antibiotics ENRO, GAM, and TYL, because sufficient strains belonging to the WT or non-WT populations were available. These antimicrobials are critically important for both human and veterinary medicine, according to the World Health Organization (WHO) and the World Organization for Animal Health (OIE). Therefore, increased knowledge on resistance mechanisms is highly relevant (25, 26).

In this GWAS analysis, we identified several previously reported mutations for ENRO resistance in M. bovis (GyrA, Ser83Phe and Glu87Gly/Val; ParC, Ser80Ile, Ser81Pro, and Asp84Asn/Tyr/Gly/Val), supporting the relevance of the output obtained (27–30). In addition, a new genetic marker in ParC (Asp79Asn) was identified and associated with acquired ENRO resistance in M. bovis. This mutation was previously observed in clinical Mycoplasma synoviae isolates and *in vitro* mutated Mycoplasma agalactiae isolates (31, 32). No mutations in the GyrB protein were associated with the phenotypes (data not shown), which was expected because such mutations are described to be associated with evolutionary mutations for which we corrected by implementing the phylogenetic tree (11, 27). Any single GyrA mutation (Ser83Phe or Glu87Gly/Val) was observed in strains with MIC values of 0.5 and 1 μg/ml, as described previously for Ser83Phe in M. bovis isolates from Israel (27). Although these strains still belonged to the WT population, in accordance with an ECOFF of >1 μg/ml, the isolates were all on the right-hand side of the normal distribution and thus close to the ECOFF. In isolates from Israel, an additional mutation in Asp84Asn (ParC) was necessary to obtain resistance (MICs of >2 μg/ml) (27), which supports our findings. Mutations in Glu87 (GyrA) were previously demonstrated only after *in vitro* selection and were thought to have no impact on resistance (28, 33). In our study, however, the Glu87Gly/Val mutation was associated with elevated MIC values (Mb225, 2 μg/ml; Mb134, 4 μg/ml) when it cooccurred with a ParC (Aps84) mutation. It was previously reported that a mutation at the same position (Asp84Asn) resulted in a 2-fold increase of the MIC (27, 28, 33), which possibly explains the increased MIC value associated with the Glu87 mutation. The effects on MIC values of other mutations at this location (Asp84Tyr/Gly) have not yet been determined (33). Therefore, further research is necessary to determine whether mutations at this location alone could result in resistance. Hata and coworkers concluded that single mutations in *parC* do not result in lower susceptibility (29). In our study, however, one isolate (Mb184) contained the single mutation at Asp84 in ParC with a non-WT phenotype (MIC of 2 μg/ml). Other resistance mechanisms might have been involved, as efflux pumps resulting in resistance to fluoroquinolones were identified in Mycoplasma hominis (34), which cannot be evaluated with the current study approach. In one isolate, the highest MIC values (≥32 μg/ml) were obtained for the combination of Ser83Phe and Ser80Ile, which is in line with mutations identified in M. bovis isolates from France, Japan, and Spain (11, 29, 30, 33).

The GWAS analysis suggested that the visually estimated ECOFF value of >2 μg/ml should be lowered to >1 μg/ml. However, some isolates containing only a mutation in Ser83 (GyrA) were not identified as non-WT by the phenotypic AST but were close to the ECOFF. To include these strains in the non-WT population, the ECOFF should be shifted toward >0.5 μg/ml or even >0.25 μg/ml, but that would result in an important number of strains being falsely categorized as non-WT (59 isolates). Therefore, an ECOFF of >1 μg/ml currently seems to be the golden mean but is clearly not perfect. Therefore, molecular methods may be superior to the phenotypic AST as a more straightforward early warning tool for the detection of emerging AMR in surveillance programs. The use of fluoroquinolones is already restricted in food-producing animals in various parts of the world, and it could be recommended to completely avoid the use of ENRO when at least one ENRO-associated mutation is found, even if phenotypic AST shows susceptibility. This is because more selection pressure could result in additional mutations and increased MIC values, as shown previously in an *in vitro* setting for M. agalactiae (35).

All non-WT isolates for the 16-membered-ring macrolide TIL contained the G748A mutation in domain II of both 23S rRNA alleles, which was also observed in previous studies (15, 19, 29, 36). An additional mutation at position 2058 was associated with GAM (15-membered ring) and TYL (16-membered-ring) resistance in our GWAS. This combination of mutations was also observed in previous TYL- and TIL-resistant isolates (16, 37, 38). The mutation at A2058 was associated previously with macrolide and lincosamide resistance in M. bovis, while only an association with lincosamide resistance could be identified in clinical isolates from Spain (11, 36). In our study, the Gln93His mutation in the L22 protein was observed in 73% of all isolates. This is a proportion similar to that reported by Lerner et al. and is below the 100% incidence reported by Kinnear et al. (15, 37). The C2611G mutation was identified in our study but was not associated with a resistance phenotype, which is in line with observations in M. pneumoniae (18). The A2062 and G2506A mutations were previously suggested to be linked to FLOR and pleuromutilin (e.g., TIA) resistance in M. bovis (28, 29). Although these mutations were identified in some of the currently investigated M. bovis isolates, no clear association with phenotypic resistance against any of the tested antimicrobials was identified. The associations between other mutations and macrolide resistance of M. bovis were nonconclusive (data not shown). This could be due to a less clear bimodal MIC distribution in the investigated population, resulting in difficulties in properly distinguishing WT and non-WT isolates from each other, as observed for the fluoroquinolones. This could be a result of mutations with minimal effects on MIC values, sampling bias (e.g., insufficient strains exploiting the same resistance mechanism), or a macrolide resistance mechanism that cannot been explained using current genetic approaches (14, 19). For example, alternative resistance mechanisms, such as target site modification by methylation, have been described for macrolides in Streptomyces fradiae (39). The potential role of horizontal chromosomal transfer or reshuffling of the *Mycoplasma* genome can also contribute to AMR, as was shown *in vitro* for ENRO resistance (35, 40). Whether this was the case in our samples should be addressed in future studies. Analogous mutations have been identified in M. bovis but have not yet been associated with AMR (19). Another macrolide AMR mechanism that was not fully investigated in the current research is the efflux of the drug by ABC transporters. While multiple (ABC-type) efflux pump genes were identified in all 95 high-quality M. bovis genomes (data not shown), their causal link with macrolide resistance still has to be confirmed using targeted mutagenesis, efflux pump inhibitors, or gene expression analyses using RNA sequencing. For M. pneumoniae, however, it has been shown that efflux pumps (possibly ABC type) are involved in resistance to macrolides (41). Cross-resistance with other antimicrobials not included in this study, such as lincosamides, may also be a likely explanation. Cross-resistance between the macrolides and lincosamides is frequently described for *Mycoplasma* species, because both classes of molecules bind to domain V of the 23S rRNA and the L22 ribosomal protein (28, 42).

When insufficient numbers of strains belong to the (non-)WT population (e.g., OTC, DOX, FLOR, TIL, GEN, and TIA), a GWAS analysis is not successful or renders inconclusive outputs. Therefore, the genetic profiling of AMR for those antimicrobial agents is limited to the detection of previously described mutations available in the literature. For OTC and DOX, several mutations previously associated with tetracycline resistance were observed, although the mutation at site 1058 that was previously described in France and Japan was not observed in this study (29, 38, 43). Alterations at position 1192 in one or two alleles were also previously associated with spectinomycin resistance (28, 29) but could not be confirmed in this study because spectinomycin was not included in the phenotypic AST. Because PG45 was the only strain lacking all of the investigated mutations, it is possible that all currently investigated strains acquired resistance to the tetracyclines to some extent, except for PG45. More isolates with a broader range of MIC values are required to identify the association between observed mutations and the (non-)WT population or elevated MIC values.

In the case of GEN, only one strain was classified as non-WT and was the only strain showing mutations (A1408G and G1488A) in either one or both 16S rRNA alleles. Due to their approximate localization to the known GEN-binding region of the 16S rRNA, both mutations are suggested to contribute to acquired GEN resistance. Whether these mutations confer higher GEN MIC values and, if so, whether both mutations are required or only one is sufficient to show a GEN resistance phenotype should be addressed in further research.

Investigating the genome by GWAS for nonsynonymous mutations associated with resistance can clarify whether ECOFFs have been selected appropriately. The present study showed that determination of the ECOFF with the visual estimation method resulted in the best agreement between the antimicrobial-resistant phenotype and the genotype for the antimicrobials that had a clear bimodal distribution. However, it also showed that statistical methods can be of great help in cases of truncated distributions (“tailing”), which are frequently observed for step-by-step resistance mechanisms, such as the fluoroquinolones (ECOFF visual estimation, >2 μg/ml; statistical methods, >1 μg/ml). The ECOFF is a very relevant tool for AMR surveillance and enables detection of resistance development in a population because it allows detection of small changes in comparison with the WT population (44). Although the ECOFF is a good (although not perfect) indicator for determination of acquired resistance, it should be kept in mind that translating ECOFFs to clinical outcomes is discouraged (44), since these values do not take host and environmental factors into account. To clinically interpret MIC values and associate these with mutations in the genome, CBPs for M. bovis should be available first. Only then can the concordance between whole-genome sequences and CBPs be assessed.

This study showed Nanopore sequencing as a rapid new tool to readily determine acquired AMR and to support evaluation of ECOFF values in M. bovis. Since conventional identification and AST for *Mycoplasma* species are quite time-consuming, the current approach allows shortening of the present sample-to-result workflow. Although pre-enriched samples were used, implementing Nanopore-based approaches immediately with field samples should be a reachable future goal to make identification and AST data for various species readily available. Using GWAS, we were able to reveal genetic markers associated with acquired AMR of M. bovis for critically important antibiotics of the fluoroquinolone and macrolide families. By using data generated in these kind of analyses, M. bovis field strains can be classified as WT or non-WT in a rapid and objective way, which is not always possible with current growth-dependent methods and the lack of widely used ECOFFs or CBPs. Therefore, rapid Nanopore sequencing may help in antimicrobial decision-making when facing an M. bovis outbreak. The applicability and output can even be broadened by expanding the input of the GWAS analysis with additional phenotypic and genomic information on (non-)WT isolates from different M. bovis populations and different antimicrobial agents.

## MATERIALS AND METHODS

### Mycoplasma bovis collection and identification.

One hundred M. bovis isolates obtained from Belgian cattle between 2014 and 2019 were collected and described in a previous study (13). Briefly, the M. bovis strains were isolated from diagnostic samples (nasal and ear swab samples, bronchoalveolar lavage fluid samples, milk samples, joint fluid samples, and abdominal fluid samples). All samples were cultured on a selective/indicative agar plate as described previously (45) and were subsequently identified with matrix-assisted laser desorption ionization–time of flight mass spectrometry (MALDI-TOF MS) (46). The isolates were stored at −80°C until phenotypic AST, and then aliquots were stored at −20°C until Nanopore sequencing was performed on freshly grown cultures.

### Phenotypic AST and interpretation.

The EUCAST Subcommittee recommends the ECOFF value as the primary comparator for identifying an association between genotype (from whole-genome sequencing data) and phenotype (14). The ECOFF separates M. bovis isolates belonging to the WT population from those with acquired resistance (non-WT) based on MIC values. In a previous study, MICs for M. bovis isolates and M. bovis PG45 were obtained with broth microdilution assays for tetracyclines (OTC and DOX), macrolides (TIL, TYL, and GAM), FLOR, GEN, ENRO, and TIA, using custom-made 96-well U-bottomed Sensititre microplates (Thermo Fisher Scientific), resulting in different ECOFF values depending on the method used to determine them (13). In the present study, the different ECOFF values obtained by visual estimation and two statistical methods (NRI and ISM [95/99%]) were explored to determine which ECOFF is best suited for the GWAS on M. bovis. Because no ECOFF could be determined for TIL, isolates with MICs of ≥32 μg/ml were categorized as the non-WT population, as previously suggested by Lerner et al. (37).

### Generation of high-quality and complete Mycoplasma bovis genome assemblies with Nanopore sequencing.

Total DNA of 100 recent M. bovis field isolates was extracted and subjected to whole-genome long-read Nanopore sequencing as described previously (24). Sequencing was performed using native DNA sequencing with the rapid barcoding sequencing kit (SQK-RBK004; Oxford Nanopore Technologies [ONT]). A total of 12 strains per run were sequenced on an R9.4.1 flow cell (ONT) using a MinION device. In each sequencing run, the M. bovis PG45 type strain (ATCC 25523) was included as a positive quality control, and mock-inoculated broth was used as a negative control. Raw data (fast5 files) were collected with the MinKnow software (v.3.6.5; ONT) and used in downstream bioinformatic analyses. Mycoplasma bovis genomes were assembled as described before. Raw read files were base called using a M. bovis-specific trained base-calling model in Bonito (v.0.2.2; ONT) to generate high-quality and reliable M. bovis sequences (23). The resulting reads were assembled into genomes using Canu (v.1.9) (47) and Medaka (v.1.0.0; ONT). Final genome assemblies were annotated using the Prokka rapid prokaryotic genome annotation pipeline (v.1.14.6) (48), and the absence of plasmids was verified by contig evaluation and Abricate (v.1.0.1) (https://github.com/tseemann/abricate) (49).

### GWAS analysis.

First, we assessed the quality of all M. bovis genomes to ensure only high-quality and complete genomes were included in downstream GWAS analysis. Only genomes with sufficient median depth (>30×) and genome completeness (at least 224/226 marker genes [>99%]) were used. Genome quality control was performed using QUAST (v.5.0.2) (50) and CheckM (v.1.1.0) (51) with the *Mycoplasma* spp. (*n* = 226 markers from 83 genomes) gene marker set. When all gene markers were present, a genome was considered 100% complete. Contamination was assessed using Kraken2 classification (v2.0.9-beta) (52) of contigs with the k2_pluspf_20200919 database. Contaminating contigs, as well as duplicated contigs, were removed on the basis of their size and effect on completeness.

A k-mer-based GWAS analysis was performed to link phenotypes to genotypes. To this end, DBGWAS software (v.0.5.4) (20) was used with default settings. The DBGWAS algorithm relies on extended k-mer searches based on compacted De Bruijn graphs to associate genetic variants with clear phenotypes. First, a list was generated to link genotypes to phenotypes by categorizing the genomes as WT (designated 0), non-WT (designated 1), or undefined (designated not available [NA]) if no phenotypic data were available. This was done for each antimicrobial drug tested, using the three ECOFF methods mentioned above, and the result was given as input along with a phylogenetic tree of all genomes generated through CSIPhylogeny, using the M. bovis PG45 type strain genome (GenBank accession number NC_014760) as reference. The final DBGWAS visualization output was evaluated by significance (*P* and *q* values), annotation, and allele frequencies of each phenotypic category. Subsequently, designated “suspicious” gene targets were extracted from the annotated genomes, aligned using MAFFT (v.4.471) (53), manually curated, and analyzed for nonsynonymous protein or nucleotide mutations in protein-coding sequences or rRNAs, respectively. A similar strategy was used for previously published genetic markers if insufficient phenotypic variation (as for TIL, tetracyclines, and GEN) prohibited the GWAS approach. Results were visualized using Interactive Tree of Life (iTOL) (v.5.7) (54).

### Data availability.

All M. bovis consensus genomes are available in the NCBI GenBank database under BioProject accession number PRJNA639688. Accession numbers and sequencing summaries can be found in File S1 in the supplemental material. Final DBGWAS analysis output links are available upon request.
