# Supplementary material for: Genome-Wide Association Study Reveals Genetic Markers for Antimicrobial Resistance in Mycoplasma bovis
Source: Microbiol Spectr. 2021 Oct 6;9(2):e00262-21. doi: 10.1128/Spectrum.00262-21 (PMC8510175; doi:10.1128/Spectrum.00262-21)
Supplement: SUPPLEMENTAL FILE 1 — Supplemental material. Download SPECTRUM00262-21_Supp_1_seq8.pdf, PDF file, 0.8 MB [file spectrum00262-21_supp_1_seq8.pdf]

Supplementary Table 1. Quality assessment and accesion numbers of all sequenced *Mycoplasma bovis* genomes.

Strain	Completeness	Genome size	Contigs	Longest contig	Acc. Num.	Coverage	Biosample Acc.	Assembly Acc.	Link
AR3	85,24	1143132	4	1072958	CP058431	1565	SAMN15246608	GCA_016452465.1	www.ncbi.nlm.nih.gov/nuccore/CP058431
AR4	100	1075398	2	1066368	CP058485	1520	SAMN15246554	GCA_016453425.1	www.ncbi.nlm.nih.gov/nuccore/CP058485
Mb1	100	1092772	1	1092772	CP058448	953	SAMN15246591	GCA_016452765.1	www.ncbi.nlm.nih.gov/nuccore/CP058448
Mb112	100	1114453	5	1016388	CP058508	421	SAMN15246531	GCA_016453845.1	www.ncbi.nlm.nih.gov/nuccore/CP058508
Mb110	100	1148388	4	1081967	CP058436	1753	SAMN15246603	GCA_016452545.1	www.ncbi.nlm.nih.gov/nuccore/CP058436
Mb103	100	1112304	3	1059282	CP058509	150	SAMN15246530	GCA_016453865.1	www.ncbi.nlm.nih.gov/nuccore/CP058509
Mb109	100	1167597	14	525705	CP058460	500	SAMN15246579	GCA_016452965.1	www.ncbi.nlm.nih.gov/nuccore/CP058460
Mb108	100	1378273	13	1078802	CP058437	1455	SAMN15246602	GCA_016452565.1	www.ncbi.nlm.nih.gov/nuccore/CP058437
Mb116	100	1154117	5	651481	CP058507	346	SAMN15246532	GCA_016453825.1	www.ncbi.nlm.nih.gov/nuccore/CP058507
Mb140	100	1142951	4	1076205	CP058506	97	SAMN15246533	GCA_016453805.1	www.ncbi.nlm.nih.gov/nuccore/CP058506
Mb134	100	1202632	5	1101582	CP058458	320	SAMN15246581	GCA_016452945.1	www.ncbi.nlm.nih.gov/nuccore/CP058458
Mb120	100	1365230	12	1076880	CP058425	578	SAMN15246614	GCA_016452365.1	www.ncbi.nlm.nih.gov/nuccore/CP058425
Mb147	100	1131370	3	1084848	CP058451	1023	SAMN15246588	GCA_016452805.1	www.ncbi.nlm.nih.gov/nuccore/CP058451
Mb130	100	1262003	7	1073042	CP058486	346	SAMN15246553	GCA_016453445.1	www.ncbi.nlm.nih.gov/nuccore/CP058486
Mb143	100	1186297	6	997113	CP058521	1236	SAMN15246518	GCA_016454085.1	www.ncbi.nlm.nih.gov/nuccore/CP058521
Mb141	100	1246635	8	911375	CP058505	159	SAMN15246534	GCA_016453785.1	www.ncbi.nlm.nih.gov/nuccore/CP058505
Mb149	100	1031892	4	843530	CP058417	436	SAMN15246622	GCA_016452205.1	www.ncbi.nlm.nih.gov/nuccore/CP058417
Mb157	98,5	1105387	4	911188	CP058504	32	SAMN15246535	GCA_016453765.1	www.ncbi.nlm.nih.gov/nuccore/CP058504
Mb158	100	1125718	3	1042103	CP058502	1317	SAMN15246537	GCA_016453745.1	www.ncbi.nlm.nih.gov/nuccore/CP058502
Mb162	100	1145144	7	997807	CP058501	1366	SAMN15246538	GCA_016453725.1	www.ncbi.nlm.nih.gov/nuccore/CP058501
Mb169	100	1076860	1	1076860	CP058500	923	SAMN15246539	GCA_016453705.1	www.ncbi.nlm.nih.gov/nuccore/CP058500
Mb152	100	1378177	12	1092208	CP058447	924	SAMN15246592	GCA_016452745.1	www.ncbi.nlm.nih.gov/nuccore/CP058447
Mb168	100	1185698	7	1080189	CP058445	603	SAMN15246594	GCA_016452705.1	www.ncbi.nlm.nih.gov/nuccore/CP058445
Mb166	100	1332416	11	1123714	CP058446	229	SAMN15246593	GCA_016452725.1	www.ncbi.nlm.nih.gov/nuccore/CP058446
Mb172	100	1150654	5	1064870	CP058450	240	SAMN15246589	GCA_016452785.1	www.ncbi.nlm.nih.gov/nuccore/CP058450
Mb175	82,25	1009450	1	1009450	CP058429	1213	SAMN15246610	GCA_016452425.1	www.ncbi.nlm.nih.gov/nuccore/CP058429
Mb174	100	1106784	3	1035531	CP058499	1443	SAMN15246540	GCA_016453685.1	www.ncbi.nlm.nih.gov/nuccore/CP058499
Mb179	100	1117840	6	1017061	CP058498	1415	SAMN15246541	GCA_016453665.1	www.ncbi.nlm.nih.gov/nuccore/CP058498
Mb180	100	1170763	5	1080712	CP058497	1687	SAMN15246542	GCA_016453645.1	www.ncbi.nlm.nih.gov/nuccore/CP058497
Mb182	100	1206875	5	1056703	CP058443	280	SAMN15246596	GCA_016452665.1	www.ncbi.nlm.nih.gov/nuccore/CP058443
Mb183	100	1182764	11	931551	CP058442	498	SAMN15246597	GCA_016452645.1	www.ncbi.nlm.nih.gov/nuccore/CP058442
Mb184	100	1919832	20	1123709	CP058434	1062	SAMN15246605	GCA_018136475.1	www.ncbi.nlm.nih.gov/nuccore/CP058434
Mb193	100	1207916	5	1084836	CP058457	765	SAMN15246582	GCA_016452925.1	www.ncbi.nlm.nih.gov/nuccore/CP058457
Mb185	100	1425943	20	1001432	CP058452	786	SAMN15246587	GCA_016452825.1	www.ncbi.nlm.nih.gov/nuccore/CP058452
Mb188	100	1298582	12	1055898	CP058466	1108	SAMN15246573	GCA_016453085.1	www.ncbi.nlm.nih.gov/nuccore/CP058466
Mb194	100	1137938	2	1091976	CP058444	398	SAMN15246595	GCA_016452685.1	www.ncbi.nlm.nih.gov/nuccore/CP058444
Mb196	100	1343469	22	986623	CP058419	380	SAMN15246620	GCA_016452245.1	www.ncbi.nlm.nih.gov/nuccore/CP058419

Mb201	100	1114350	4	1022634	CP058435	805	SAMN15246604	GCA_016452525.1	www.ncbi.nlm.nih.gov/nuccore/CP058435
Mb200	100	1447813	18	799017	CP058416	246	SAMN15246623	GCA_016452185.1	www.ncbi.nlm.nih.gov/nuccore/CP058416
Mb206	100	1299193	10	536541	CP058439	124	SAMN15246600	GCA_016452585.1	www.ncbi.nlm.nih.gov/nuccore/CP058439
Mb208	87,55	1080091	2	1058586	CP058454	472	SAMN15246585	GCA_016452865.1	www.ncbi.nlm.nih.gov/nuccore/CP058454
Mb214	100	1084374	4	1019631	CP058484	2199	SAMN15246555	GCA_016453405.1	www.ncbi.nlm.nih.gov/nuccore/CP058484
Mb209	100	1131912	4	1086585	CP058430	979	SAMN15246609	GCA_016452445.1	www.ncbi.nlm.nih.gov/nuccore/CP058430
Mb216	100	1104572	1	1104572	CP058432	1123	SAMN15246607	GCA_016452485.1	www.ncbi.nlm.nih.gov/nuccore/CP058432
Mb217	100	1264802	10	1036863	CP058465	703	SAMN15246574	GCA_016453065.1	www.ncbi.nlm.nih.gov/nuccore/CP058465
Mb218	100	1158040	7	760990	CP058491	353	SAMN15246548	GCA_016453545.1	www.ncbi.nlm.nih.gov/nuccore/CP058491
Mb219	100	1163136	4	1068778	CP058490	361	SAMN15246549	GCA_016453525.1	www.ncbi.nlm.nih.gov/nuccore/CP058490
Mb222	100	1058799	1	1058799	CP058496	1003	SAMN15246543	GCA_016453625.1	www.ncbi.nlm.nih.gov/nuccore/CP058496
Mb227	100	1045944	4	970195	CP058420	482	SAMN15246619	GCA_016452265.1	www.ncbi.nlm.nih.gov/nuccore/CP058420
Mb225	100	1208632	7	521336	CP058483	1424	SAMN15246556	GCA_016453385.1	www.ncbi.nlm.nih.gov/nuccore/CP058483
Mb231	100	1168880	5	1073408	CP058495	697	SAMN15246544	GCA_016453605.1	www.ncbi.nlm.nih.gov/nuccore/CP058495
Mb229	100	1301626	8	794201	CP058489	433	SAMN15246550	GCA_016453505.1	www.ncbi.nlm.nih.gov/nuccore/CP058489
Mb237	100	1259587	8	515716	CP058426	797	SAMN15246613	GCA_016452385.1	www.ncbi.nlm.nih.gov/nuccore/CP058426
Mb239	100	1083424	2	1062432	CP058418	294	SAMN15246621	GCA_016452225.1	www.ncbi.nlm.nih.gov/nuccore/CP058418
Mb240	100	1093675	6	987968	CP058441	188	SAMN15246598	GCA_016452625.1	www.ncbi.nlm.nih.gov/nuccore/CP058441
Mb241	100	1114212	5	1013941	CP058428	718	SAMN15246611	GCA_016452405.1	www.ncbi.nlm.nih.gov/nuccore/CP058428
Mb258	100	1156358	3	946158	CP058421	471	SAMN15246618	GCA_016452285.1	www.ncbi.nlm.nih.gov/nuccore/CP058421
Mb252	100	1199475	6	1014087	CP058422	621	SAMN15246617	GCA_016452305.1	www.ncbi.nlm.nih.gov/nuccore/CP058422
Mb267	99,62	1134336	8	645391	CP058440	430	SAMN15246599	GCA_016452605.1	www.ncbi.nlm.nih.gov/nuccore/CP058440
Mb268	100	1113204	2	1075737	CP058433	912	SAMN15246606	GCA_016452505.1	www.ncbi.nlm.nih.gov/nuccore/CP058433
Mb272	100	1133146	2	1092476	CP058488	753	SAMN15246551	GCA_016453485.1	www.ncbi.nlm.nih.gov/nuccore/CP058488
Mb274	100	1165004	5	1092984	CP058494	873	SAMN15246545	GCA_016453585.1	www.ncbi.nlm.nih.gov/nuccore/CP058494
Mb275	100	1223989	8	585771	CP058493	570	SAMN15246546	GCA_016453565.1	www.ncbi.nlm.nih.gov/nuccore/CP058493
Mb276	99,25	1142548	8	772362	CP058487	370	SAMN15246552	GCA_016453465.1	www.ncbi.nlm.nih.gov/nuccore/CP058487
Mb287	100	1126948	1	1126948	CP058453	783	SAMN15246586	GCA_016452845.1	www.ncbi.nlm.nih.gov/nuccore/CP058453
Mb32	100	1220230	6	673525	CP058511	86	SAMN15246528	GCA_016453905.1	www.ncbi.nlm.nih.gov/nuccore/CP058511
Mb37	100	1101451	2	1084346	CP058423	231	SAMN15246616	GCA_016452325.1	www.ncbi.nlm.nih.gov/nuccore/CP058423
Mb49	100	1113021	1	1113021	CP058524	1044	SAMN15246515	GCA_016454145.1	www.ncbi.nlm.nih.gov/nuccore/CP058524
Mb50	99,62	1341270	13	1020093	CP058523	705	SAMN15246516	GCA_016454125.1	www.ncbi.nlm.nih.gov/nuccore/CP058523
Mb51	100	1255644	4	1130629	CP058456	311	SAMN15246583	GCA_016452905.1	www.ncbi.nlm.nih.gov/nuccore/CP058456
Mb59	100	1315295	10	1007548	CP058455	907	SAMN15246584	GCA_016452885.1	www.ncbi.nlm.nih.gov/nuccore/CP058455
Mb6	99,25	1119055	4	1055554	CP058513	148	SAMN15246526	GCA_016453945.1	www.ncbi.nlm.nih.gov/nuccore/CP058513
Mb7	100	1087220	4	1026811	CP058512	163	SAMN15246527	GCA_016453925.1	www.ncbi.nlm.nih.gov/nuccore/CP058512
Mb75	100	1122584	3	1078927	CP058424	2005	SAMN15246615	GCA_016452345.1	www.ncbi.nlm.nih.gov/nuccore/CP058424
Mb98	100	1285588	8	420169	CP058510	253	SAMN15246529	GCA_016453885.1	www.ncbi.nlm.nih.gov/nuccore/CP058510
PG45_10	100	1019609	1	1019609	CP058415	1737	SAMN15246624	GCA_018136415.1	www.ncbi.nlm.nih.gov/nuccore/CP058415

TO19282‹	100	1150555	3	1085822	CP058522	341	SAMN15246517	GCA_016454105.1	www.ncbi.nlm.nih.gov/nuccore/CP058522
TOVK	91,15	1215647	11	1013283	CP058464	469	SAMN15246575	GCA_016453045.1	www.ncbi.nlm.nih.gov/nuccore/CP058464
VK10	87,39	1162961	5	1012438	CP058480	1029	SAMN15246559	GCA_016453345.1	www.ncbi.nlm.nih.gov/nuccore/CP058480
VK11	100	1182271	3	1115809	CP058479	618	SAMN15246560	GCA_016453325.1	www.ncbi.nlm.nih.gov/nuccore/CP058479
VK12	100	1159464	4	1120839	CP058461	1533	SAMN15246578	GCA_016452985.1	www.ncbi.nlm.nih.gov/nuccore/CP058461
VK14	100	1055408	2	1032289	CP058477	881	SAMN15246562	GCA_016453285.1	www.ncbi.nlm.nih.gov/nuccore/CP058477
VK13	100	1287141	6	1125147	CP058478	357	SAMN15246561	GCA_016453305.1	www.ncbi.nlm.nih.gov/nuccore/CP058478
VK16	100	1276688	17	451653	CP058475	541	SAMN15246564	GCA_016453245.1	www.ncbi.nlm.nih.gov/nuccore/CP058475
VK22	100	1107730	1	1107730	CP058473	1150	SAMN15246566	GCA_016453205.1	www.ncbi.nlm.nih.gov/nuccore/CP058473
VK15	100	1969687	25	888690	CP058476	495	SAMN15246563	GCA_016453265.1	www.ncbi.nlm.nih.gov/nuccore/CP058476
VK24	100	1120833	7	1015321	CP058471	386	SAMN15246568	GCA_016453165.1	www.ncbi.nlm.nih.gov/nuccore/CP058471
VK19	100	1780942	28	593455	CP058474	732	SAMN15246565	GCA_016453225.1	www.ncbi.nlm.nih.gov/nuccore/CP058474
VK25	100	1213361	9	560089	CP058482	774	SAMN15246557	GCA_016453365.1	www.ncbi.nlm.nih.gov/nuccore/CP058482
VK26	100	1169535	8	783105	CP058469	445	SAMN15246570	GCA_016453145.1	www.ncbi.nlm.nih.gov/nuccore/CP058469
VK23	99,25	1999178	42	346703	CP058472	1450	SAMN15246567	GCA_016453185.1	www.ncbi.nlm.nih.gov/nuccore/CP058472
VK27	99,25	1137722	9	783979	CP058468	648	SAMN15246571	GCA_016453125.1	www.ncbi.nlm.nih.gov/nuccore/CP058468
VK3	100	1157958	8	998194	CP058520	424	SAMN15246519	GCA_016454065.1	www.ncbi.nlm.nih.gov/nuccore/CP058520
VK32_2	100	1141742	3	1081535	CP058462	1186	SAMN15246577	GCA_016453005.1	www.ncbi.nlm.nih.gov/nuccore/CP058462
VK30	99,25	1511419	24	660367	CP058467	185	SAMN15246572	GCA_016453105.1	www.ncbi.nlm.nih.gov/nuccore/CP058467
VK41	100	1104361	4	1029275	CP058463	1112	SAMN15246576	GCA_016453025.1	www.ncbi.nlm.nih.gov/nuccore/CP058463
VK5	100	1278744	10	677514	CP058519	1156	SAMN15246520	GCA_016454045.1	www.ncbi.nlm.nih.gov/nuccore/CP058519
VK6	100	1106605	3	1074537	CP058518	911	SAMN15246521	GCA_016454025.1	www.ncbi.nlm.nih.gov/nuccore/CP058518
VK7	100	1114769	7	998710	CP058517	686	SAMN15246522	GCA_016454005.1	www.ncbi.nlm.nih.gov/nuccore/CP058517
VK8	100	1129102	3	1067173	CP058516	645	SAMN15246523	GCA_016453985.1	www.ncbi.nlm.nih.gov/nuccore/CP058516
VK9	100	1161869	5	1056461	CP058515	353	SAMN15246524	GCA_016453965.1	www.ncbi.nlm.nih.gov/nuccore/CP058515
**Mean**	**99,31**	**1196908,71**	**6,92**	**964184,72**					
**Stdev**	**2,83**	**168825,14**	**6,27**	**182255**					

Genomes excluded from GWAS analyses are indicated in red.

Supplemental file 2. Results of GWAS on 96 *M. bovis* isolates based on different ECOFF methods

			**Visual estimation**			**WT Vs. nWT**	**NRI**			**WT Vs. nWT**
**Class**	**Antimicrobial**	**Gene**	**p-value**	**q-value**	**Significance**		**p-value**	**q-value**	**Significance**	
**Fluoroquinolone**	**Enrofloxacin**	***ParC***	2,07E-50	2,06E-46	40 unitigs (14 significants)	85/8	7,38E-43	7,35E-39	40 unitigs (12 significants)	83/10
	**Enrofloxacin**	***GyrA***	4,11E-19	8,19E-16	23 unitigs (4 significants)	85/8	5,59E-18	1,11E-14	23 unitigs (4 significants)	83/10
**Macrolide**	**Tilmicosin**		*ND*	*ND*	*ND*	*ND*				25/70
	**Tylosin**	**23S rRNA**	6,90E-13	6,87E-09	24 unitigs (4 significants)	46/50	6,90E-13	6,87E-09	24 unitigs (4 significants)	46/50
	**Tylosin**	**rOperon**	1,28E-06	4,24E-03	2452 unitigs (139 significants)	46/50	1,28E-06	4,24E-03	2452 unitigs (139 significants)	46/50
	**Gamithromycin**	**23S rRNA**	6,85E-11	6,83E-07	24 unitigs (3 significants)	53/43	2,70E-12	2,69E-08	24 unitigs (3 significants)	56/40
	**Gamithromycin**	**rOperon**	4,12E-06	1,37E-02	4929 unitigs (226 significants)	53/43	6,70E-07	1,33E-03	4833 unitigs (183 significants)	56/40

			**ECOFFinder95%**			**WT Vs. nWT**	**ECOFFinder99%**			**WT Vs. nWT**
**Class**	**Antimicrobial**	**Gene**	**p-value**	**q-value**	**Significance**		**p-value**	**q-value**	**Significance**	
**Fluoroquinolone**	**Enrofloxacin**	***ParC***	7,38E-43	7,35E-39	40 unitigs (12 significants)	83/10	2,07E-50	2,06E-46	40 unitigs (14 significants)	85/8
	**Enrofloxacin**	***GyrA***	5,59E-18	1,11E-14	23 unitigs (4 significants)	83/10	4,11E-19	8,19E-16	23 unitigs (4 significants)	85/8
**Macrolide**	**Tilmicosin**		*ND*	*ND*	*ND*	*ND*	*ND*	*ND*	*ND*	*ND*
	**Tylosin**	**23S rRNA**	*ND*	*ND*	*ND*	*ND*	*ND*	*ND*	*ND*	*ND*
	**Tylosin**	**rOperon**	*ND*	*ND*	*ND*	*ND*	*ND*	*ND*	*ND*	*ND*
	**Gamithromycin**	**23S rRNA**	*ND*	*ND*	*ND*	*ND*	*ND*	*ND*	*ND*	*ND*
	**Gamithromycin**	**rOperon**	*ND*	*ND*	*ND*	*ND*	*ND*	*ND*	*ND*	*ND*
